# The Role of Social Determinants in Mental Health and Resilience After Disasters: Implications for Public Health Policy and Practice

**DOI:** 10.3389/fpubh.2021.658528

**Published:** 2021-05-19

**Authors:** Wanying Mao, Vincent I. O. Agyapong

**Affiliations:** Department of Psychiatry, Faculty of Medicine and Dentistry, University of Alberta, Edmonton, AB, Canada

**Keywords:** mental health, resilience, disaster, social determinant, public health policy

## Abstract

In this general literature review, we will explore the impacts and contribution of social determinants to mental health and resiliency following both natural and man-made disasters. Natural disasters, such as wildfires, earthquakes, tsunamis, and hurricanes, as well as man-made disasters, such as civil wars, have been known to inflict significant damage to the mental health of the victims. In this paper, we mainly explore some most studied vulnerability and protective social determinant factors such as gender, age, ethnicity, socials support and socioeconomic status for the mental health and resiliency in survivors of such disasters. Several other possible factors such as previous trauma, childhood abuse, family psychiatric history, and subsequent life stress that were explored by some studies were also discussed. We conducted a literature search in major scientific databases, using keywords such as: mental health, social determinants, disasters, wildfires, earthquakes, terrorist attacks, and resilience. We discuss the implications for public health policy and practice.

## Introduction

According to the World Health Organization (WHO), mental health is defined as the successful performance of an individual's mental capabilities. Mental health is a state of well-being in which each person realizes their potential, can manage the changes and everyday stresses of life, can work efficiently and productively, and is capable to contribute to his or her own community (1). Mental health includes emotional, psychological, and social well-being. It is also essential to recognize that the absence of a mental disorder does not indicate sound mental health (2, 3).

There has been increased recognition that social and economic circumstances affect people's mental health, and that natural and man-made disasters can also impact the resiliency of one's mental health. Social determinants include the condition in which people were born, live, and work; their age; ethnic status, and so on. They include factors such as social-economic status, educational attainment, neighborhood, and physical environment, employment, social support networks, as well as access to health care (4), which are in turn determined by a wider amount of forces, such as macroeconomics, environment, and politics (5).

As large-scale events, disasters are often unexpected (6, 7). They disrupt the normal conditions of existence, can cause death, trauma, and the destruction of property that can exceed the affected communities' capacity to adjustment (8). Each year, disasters affect millions of people worldwide. Averagely, at least one disaster happens each day around the world, and because of climate change and the growing population density, the frequency and impact of disasters keep staying at a high level (9). In 2017, over 11,000 people died or disappeared due to disasters, while millions were left homeless. In 2018, more than 13,500 people lost their lives or went missing from disasters, with damages totaling $165 billion of economic losses (10). In 2019, 11,000 people around the world perished or were missing in natural and human-made disasters (11).

Studies usually classify disasters into three different types: natural disaster, such as earthquake; man-made non-intentional technological disaster, such as the nuclear accident at Chernobyl; man-made intentional acts, for example, the mass violence and terrorism as seen on September 11, 2001 World Trade Center (WTC) attacks (7, 12). Although the area remains controversial to study, some research indicates that man-made disasters and mass violence incidents tend to create greater mental and psychological impacts on survivors than natural disasters (7, 13). However, considering the wider picture of human health, it might be more critical and useful to focus on the traumatic event's characteristics, instead of the cause, since different types of disasters can have many similarities in terms of impact and outcome. Moreover, like the events in Fukushima, Japan, 2011 demonstrate, some disasters can consist of both natural and technological disaster, meaning some disasters can be multi-type (9). Accordingly, in this review paper, we go beyond categorizing disasters by cause and instead highlight the aspects of the disaster experience that can be associated with the psychological and mental health problems of these events.

The psychological consequences of natural and man-made disasters have been studied intensively in the past 20 years (14), with research also exploring the mental health impacts on victims of disasters, such as Hurricane Katrina in 2005, Hurricane Sandy in 2012, the Fukushima nuclear disaster in 2011, and the terror attack on the World Trade Center in 2001 (15). Studies of traumatic events often indicate that among people who have been directly affected by disasters and events of mass violence, a majority of do not develop psychopathological issue and cope well in the aftermath of disaster (16, 17). The capacity to continue functioning after encountering a disaster is common and characteristic of normal coping and adaptation mechanisms, with the phenomenon becoming known as “resilience” (18). The American Psychological Association (APA) defined resilience as the “process of adapting well in the face of adversity, trauma, tragedy, threats or even significant sources of stress” (19). However, not everyone can function well and demonstrate resilience after a traumatic event, with some survivors experiencing psychological impairments, while a small number of these develop more serious forms of mental disorder (7) such as major depression, generalized anxiety, and post-traumatic stress disorder (PTSD). These extreme outcomes may last for extended periods following the disaster and may even be present for the rest of their lives (15).

## Study Aims

The growing recognition of mental health issues highlights the need to develop an understanding of what makes some people more vulnerable to be negatively impacted by disasters. A significant amount of study has led to the recognition of a series of social, physical, and economic factors referred to as the potential causes of mental health issues after disaster. In this paper, we review the current state of the field of disaster-related mental health research, with the aim of exploring the vulnerability and protective social determinant factors which impact on the mental health and resiliency in survivors of disasters. To achieve this aim, we will conduct a general literature search in major scientific databases using keywords such as: mental health, social determinants, disasters, wildfires, earthquakes, terrorist attacks, and resilience, whilst also describing challenges and limitations to our current methodologies, thus allowing for suggestions about the direction of future research.

## Methodology

This general review aims to explore the impacts and contributions of social determinants to mental health and resiliency following both natural and man-made disasters. The review was conducted through a general search of the literature on Google Scholar, PubMed, PsycINFO, SpringerLink, Taylor & Francis Online, BioMed Central, and ScienceDirect. Search terms included social determinants (e.g., gender, age, ethnicity, social supports, socioeconomic status, etc.) mental health (e.g., PTSD, depression, anxiety, resilience, etc.) and disasters (e.g., floods, earthquakes, terrorist attacks). We began the review with an examination of the definitions, origins, and types of disaster to deepen and clarify the understanding of the main concepts. We summarized the key findings in literature on the social determinants and protective factors for mental health following disasters in a tabular form. We then discussed the vulnerability and protective social determinant factors such as gender, age, ethnicity, social support, and socioeconomic status for the mental health and resiliency in survivors of such disasters by reference to existing literature. We also discussed several other possible social determinants that were studied by some researches. Thereafter, we provided a conceptual framework for the relationship between social determinants and the resiliency of mental health problems in the post-disaster period as well as discuss the policy implications. In the end, we concluded the review by examining some areas related to this field that may benefit most from future research and provided a commentary on the possible research topic for future study.

## Results

Table 1 summarize key literature on social determinants and protective factors for mental health following disasters.

**Table 1:** Summary of studies on key social determinants and protective factors for mental health following disasters.

**References**	**Study Aims**	**Methods**	**Main Findings**
Agyapong et al. (27, 91)	To assess likely prevalence of PTSD in residents of Fort McMurray 6 months after a wildfire and to determine the predictors of likely PTSD in the respondents.	A quantitative cross-sectional survey was used to collect data through self-administered paper-based questionnaires to determine likely PTSD.	• Support from family/friends following wildfires may be protective against likely PTSD, • A prior diagnosis of an anxiety disorder significantly increased risk for developing PTSD
Agyapong et al. (26)	This study describes the changes in the stress, anxiety, and depression levels of subscribers to the Text4Hope program after 6 weeks of exposure to daily supportive SMS text messages.	Self-administered web-based questionnaires were used to assess the demographic and clinical characteristics of subscribers. Perceived stress, anxiety, and depression were measured with PSS-10, GAD-7 scale, and PHQ-9 scale at baseline and 6th week time points. Moderate or high perceived stress were assessed using cutoff scores.	• At the 6-week time point, there were statistically significant reductions in mean scores on the PSS-10 and GAD-7 scales but not on the PHQ-9 scale. • There were statistically significant reductions in the prevalence rates of moderate or high stress and likely generalized anxiety disorder but not likely major depressive disorder for the group that completed both the baseline and 6-week assessments. The largest reductions in mean scores and prevalence rates were for anxiety (18.7 and 13.5%, respectively).
Agyapong et al. (139, 140)	This study describes the changes in the stress, anxiety, and depression levels of subscribers to the Text4Hope program after 3rd month of exposure to daily supportive SMS text messages.	Self-administered web-based questionnaires were used to assess the demographic and clinical characteristics of subscribers. Perceived stress, anxiety, and depression were measured with PSS-10, GAD-7 scale, and PHQ-9 scale at baseline and 3rd month time points. Moderate or high perceived stress were assessed using cutoff scores.	• After 3 months of using Text4Hope, subscribers' self-reports revealed significant (p < 0.001) mean score reductions compared with baseline on: the GAD-7 by 22.7%, PHQ-9 by 10.3%, and PSS-10 scores by 5.7%. • Reductions in inferred prevalence rates for moderate to high symptoms were also observed, with anxiety demonstrating the largest reduction (15.7%).
Brewin et al. (29)	To investigate the risk factors for posttraumatic stress disorder in trauma-exposed adults.	Meta-analyses were conducted on 14 separate risk factors for PTSD, and the moderating effects of various sample and study characteristics, including civilian/military status, were examined.	• Gender, age at trauma, and race predicted PTSD in some populations. • Education, previous trauma, and general childhood adversity predicted PTSD consistently. • Psychiatric history, reported childhood abuse, and family psychiatric history that had more uniform predictive effects
Chan et al. (88)	To examine the contribution of pre- and post-disaster social support to short- and long-term mental health after Hurricanes Katrina	This is a three-wave longitudinal study, 492 residents in the region affected by Hurricane Katrina reported levels of perceived social support and symptoms of psychological distress prior to the storm (Wave 1). One year after Hurricane Katrina (Wave 2), they reported levels of exposure, perceived social support, and symptoms of psychological distress and posttraumatic stress. The latter three variables were assessed again 4 years after the hurricane (Wave 3).	• Pre-Katrina support predicts psychological outcomes by reducing hurricane exposure. • Post-Katrina social support predicts later psychological distress but not posttraumatic stress. • Four years after Katrina, perceived social support remains lower than baseline.
Cherry et al. (59)	To examine the effects of Hurricanes Katrina and Rita on cognitive and psychosocial functioning in adults 6–14 months after the storms.	Participants were recruited from the Louisiana Healthy Aging Study (LHAS). Most were assessed during the immediate impact period and retested for this study.	• Middle-aged adults reported more storm-related stressors and greater levels of stress than the two older groups at both waves of testing.
Dai et al. (95)	To measure the prevalence rate of PTSD at follow-up and identify predictors of recovery from the PTSD diagnosis in 2000.	PTSD at follow-up was reassessed using the PTSD Checklist-Civilian version. Information on demographics, trauma-related stressors, and coping style were collected through face-to-face interviews. Logistic regression was used for data analyses.	• Individuals who had lost relatives, suffered from bodily injury, had a low level of social support, or had a negative coping style were less likely to recover from PTSD.
Galea et al. (13)	To comprehensively and systematically assessing the epidemiologic evidence about PTSD after disasters.	This is a systematic review. It limited this review to studies conducted between 1980, when PTSD was first codified as a disorder in the DSM, DSM-III, and 2003.	• Most studies have focused on adults who were direct victims of the disaster. • Women has consistently been shown to be more likely to get PTSD after disasters. • A history of prior traumas has been associated with PTSD onset across multiple studies, as has low social support and having poor relationships with family members and coworkers.
Green et al. (52)	To investigate how age, gender and parental functioning effects PTSD symptoms on Children after disaster.	179 children aged 2–15 who were exposed to the Buffalo Creek dam collapse in 1972 were rated for PTSD symptoms 2 years after the disaster. Age and gender effects and the impact of the level of exposure and parental functioning were examined according to a conceptual model addressing factors contributing to adaptation to a traumatic event.	• Fewer PTSD symptoms were found in the youngest age group. • Higher symptom levels for girls than boys. • Life threat, gender, parental psychopathology, and an irritable and/or depressed family atmosphere all contributed to the prediction of PTSD symptomatology in the children.
Hall et al. (96)	To evaluate if baseline psychological distress symptoms and changes in these symptoms were associated with changes in social resources 5 months later among treatment-seeking torture survivors residing in Kurdistan, Iraq.	This longitudinal study used the Hopkins Symptom Checklist-25, Harvard Trauma Questionnaire, and a traumatic grief measure to collect information; Locally derived scales were used to measure perceived social support related measures. Multinomial logistic regression models assessed the association between symptoms and loss or gain in social resources.	• Higher mental health symptoms have a correlation with decreased social support. • The decline of social contact was associated with increased anxiety and PTSD symptoms. • Gaining social contact was related to decreasing depression and PTSD symptoms
Irmansyah et al. (31)	To exploring the effect on mental health of direct exposure to the tsunami.	Questionnaire was collected information from 783 people aged 15 years in tsunami-affected areas of Aceh and Nias. Group comparisons, contrasting responses Internally Displaced Persons and non-IDPs, were by chi-square for frequency data and *t*-tests for ordinal or continuous data. Hierarchical multiple linear regression analyses were performed to examine the relative contributions to psychopathology of demographic variables and measures of exposure, impact and resilience.	• High rates of psychopathology were recorded in the overall sample, particularly in those who experienced more substantial post-disaster changes in life circumstances. • Higher Self-Reporting Questionnaire scores were observed among women, those with lower education, those with diminished resilience beliefs, those experiencing high scores on disaster impact, those experiencing direct exposures to the disaster
Johnson et al. (98)	This study is a longitudinal course of psychiatric sequelae of a mass shooting incident at a courthouse.	Participants were interviewed 6–8 weeks after the incident and followed up 1 year and 3 years later. DIS/DS was used to collected data. Chi-square analyses, McNemar tests, *T*-tests and Multiple regression analysis were applied to do data analysis.	• Pre-existing psychopathology was a strong indicator of those at risk for a post disaster disorder
Kessler et al. (67)	To get a comprehensive understanding about the PTSD on the general population through National Comorbidity Survey.	Modified versions of the DSM-III-R PTSD module from the Diagnostic Interview Schedule and of the Composite International Diagnostic Interview were administered to a representative national sample of 5,877 persons aged 15–54 years in the part II subsample of the National Comorbidity Survey.	• The estimated lifetime prevalence of PTSD is 7.8%. • Prevalence is elevated among women and the previously married. • The traumas most commonly associated with PTSD are combat exposure and witnessing among men and rape and sexual molestation among women. • More than one third of people with an index episode of PTSD fail to recover even after many years.
Neria et al. (6)	To systematically assess the evidence about PTSD following exposure to disasters.	A systematic search was performed. Eligible studies for this review included reports based on the DSM criteria of PTSD symptoms. The timeframe for inclusion of reports in this review is from 1980 and February 2007 when the literature search for this examination was terminated.	• Post-disaster PTSD is associated with a range of correlates including sociodemographic and background factors, event exposure characteristics, social support factors and personality traits.
North et al. (30)	To understand the coping, functioning and adjustment of rescue workers after the Oklahoma City Bombing.	The participants were the 181 firefighters who served first as rescue workers. Pre and post disaster psychiatric diagnoses were assessed with the DIS for DSM-III-R. The Disaster Supplement 9 elicited additional information in open-ended questions. Chi-square tests, Fisher's exact tests and Wilcoxon tests were performed for data analysis.	• Functional impairment was uncommon (15%) in firefighters without PTSD but common (83%) in those with this diagnosis • The only aspect of interpersonal functioning manifesting significant problems after the bombing was marital disruption. • Among these firefighters nearly 50% rates of lifetime and 25% rates of current alcohol use disorder
Orui et al. (43)	To determine whether the tsunami disaster following the Great East Japan Earthquake has influenced the national suicide rates.	The time-series analysis and the Poisson distribution test were used to compare suicide rates in the tsunami disaster-stricken areas to national averages	• In tsunami disaster-stricken areas, male suicide rates were significantly lower than the national average at the beginning and started to increase after 2 years. But not for female.
Perilla et al. (62)	To investigate how natural disasters affect the mental health of people with different ethnicity	404 residents of southern Florida were interviewed 6 months after Hurricane Andrew. The sample was composed of equal numbers of Hispanics, non-Hispanic blacks, and Caucasians. 30-item Revised Civilian Mississippi Scale were used to assess posttraumatic stress.	• Ethnic groups differed strongly in the prevalence of PTSD • Caucasian disaster victims showed the lowest rate (15%), Spanish-preferring Latinos showed the highest rate (38%), and African Americans showed a rate (23%) between these two extremes.
Rafiey et al. (58)	To compare positive mental health between elderly and young earthquake survivors.	Data of the 324 earthquake survivors were obtained from a population-based cross-sectional survey conducted in Iran, 2015. The long-term effect of earthquake was assessed using the Mental Health Continuum-Short Form questionnaire. A one-way multivariate analysis of covariance (MANCOVA) using SPSS (version 22) was used in data analysis.	• Elderly earthquake survivors showing a higher level of positive mental health compared with their younger counterparts in the wake of natural disasters suggest that advancing per se does not contribute to increasing vulnerability.
Sasaki et al. (86)	To examine whether pre-disaster social support functions can mitigate post-disaster depressive symptoms among older survivors of the 2011 Great East Japan earthquake and tsunami.	This study was a part of the Japan Gerontological Evaluation Study, which began in 2010 as a nationwide, population-based, prospective cohort study investigating the predictors of physical and psychological health in community-dwelling Japanese older adults. In the present longitudinal study, the study used panel data from two waves of the JAGES survey.	• Participants who gave and received emotional and instrumental support before the disaster were significantly less likely to develop depressive symptoms after the disaster compared to those without support
Subaiya et al. (121)	To evaluate the association between socioeconomic status (SES) and storm recovery.	The study conducted a cross-sectional survey within the Rockaways 3 weeks after the hurricane made landfall to elicit information regarding basic utilities, food access, health, relief-effort opinions, and SES. It used a modified cluster sampling method to select households with a goal of 7–10 surveys per cluster.	• Lower-income households were more likely to worry about food than higher-income households. • A post-storm trend also existed among the lower-income group toward psychological disturbances.
Tortella-Feliu et al. (28)	To identify what factors may be associated with increased or decreased risk for PTSD	Researchers conducted an umbrella review of systematic reviews and meta-analyses of risk/protective factors for PTSD and assessed and graded the evidence of the association between each factor and PTSD.	• Being female or being indigenous people of the Americas; history of physical disease and family history of psychiatric disorder, and cumulative exposure to potentially traumatic experiences, trauma severity, and being trapped during an earthquake, showed convincing evidence of an association with PTSD.

### Discussion

Exposure to disasters has been related to various mental health outcomes (7). As summarized in Table 1, many social determinants have been used to deduce what kind of people are more liable to suffer from the adverse effects of traumatic events and thus more vulnerable to mental health issues (15). The following section discusses the vulnerability factors and protective social determinant factors for mental health and resiliency in survivors of disasters. This section consists of several sub-sections, starting with gender, followed by age, ethnicity, social support, and social-economic status.

### Gender

Momsen defines gender as “the socially acquired notions of masculinity and femininity by which women and men are identified” (20) (p. 2). Accepting the gendered perspective means considering the biological and psychological differences between men and women, whilst also analyzing their experiences in relation to situations and circumstances in which they were in.

Females have consistently been regarded as one of the key factors for post-disaster mental health problems (21–25). Many researches indicate that, psychological adverse consequences of disasters, such as PTSD and depression, are usually more serious for women survivors (6, 13, 26–28). Others like Brewin et al. (29) found that factors such as gender predicted PTSD in some populations but not in others.

In a study compared different populations experienced terrorist bombings (30), using structured diagnostic interviews to research the subjects. The study investigated victims who had experienced bombings of the US Embassy in Nairobi, Kenya, and bombings of the Oklahoma City Federal Building. The results revealed that, 6 months after the Oklahoma City bombing, among the 182 survivors, 22% of males and 40% of females displayed PTSD symptoms (30). Moreover, for the 227 survivors of the Nairobi bombing, 34% of males and 49% of females were detected to have PTSD symptoms (30). Similar results can be found in Galea's publication about the epidemiology of post-traumatic stress disorder after disasters (13) as well as in a study conducted about the survivors of the earthquake and tsunami in Aceh and Nias (31). Furthermore, Bonanno and Gupta also noted that female survivors are less likely than male survivors to be resilient in the period following catastrophic events (18). This gender difference has been observed in both adults and children, as well as in developed and developing countries, whilst the differences exist regardless of the type of mental disorder (7, 32). This reflects the higher prevalence of mood and psychological disorders among women after disasters (33).

When considering the reasons behind this gender difference, men can be physically and mentally better equipped to withstand a disaster's impact (34), whilst many studies believe differences are primarily caused by the traditional gender roles as well as the existing gender inequalities (8, 34–36). Women often experience unequal access to resources and relief assistance (37, 38), for instance, they may be forced to have “transactional sex” to acquire necessities of life such as food during emergencies (39). Moreover, domestic violence and rape cases may grow after disasters (40, 41). Additionally, poor sanitary conditions in sheltered accommodation can create not only physical but mental health risks also especially to women (39, 42). For example, in 1998, after the floods in Bangladesh, young girls reported perineal rashes and urinary tract infections mainly because they were not able to access clean water to wash menstrual rags, whilst also having no place to dry the rags (8). Furthermore, another burden for women is their caregiver roles and responsibilities at home, where work of this kind may increase post-disaster and cause more stress and psychological problems (40).

However, what cannot be neglected is that there are examples of where mental health of males experiences were more adversely affected compared to females after disaster (43, 44). In the 3-year follow-up study about suicide rates in tsunami disaster-stricken areas following the Great East Japan Earthquake, with a time-series analysis and the Poisson distribution test, suicide rates in the tsunami disaster-stricken areas were compared to national averages. Orui et al. found that, in tsunami disaster-stricken areas, male suicide rates were remarkably lower than the national average during the initial post-disaster period and start to rise 2 years after (43). Besides, after decreased for seven months, the suicide rates of male in the inland areas increased to exceed the national average. By comparison, female post-disaster suicide rates did not change in both areas compared to the national average. Notably, the male suicide rates in the inland areas started to increase earlier compared to the tsunami-stricken areas, which may reflect the relative deficiency of mental healthcare services in the inland areas (43). Another study investigated farming suicides during the Victorian drought from 2001 to 2007 revealed the similar outcome (44). The study was carried out to explore whether farming suicides increased in Victoria during the prolonged drought in south eastern Australian. The results indicated that farming suicides accounted for just ~3% if Victorian suicides. In total, the number of farming suicides was 110 for the year and ranged between 11 and 19 deaths each year, increasing and decreasing inconsistently from year to year. Males taken up around 95% of farming suicides, with firearms and hanging as the most frequently used methods and the majority of the death cases happening between 30 and 59 years old (44).

### Age

Another recognized factor that affects people's mental health and resiliency following the natural and man-made disaster is their age.

According to the United Nations Convention on the Rights of the Child, children are defined as persons age at 18 and younger. Based on the statistical data, at the end of the 20th-century, disasters affected ~66.5 million children each year (45). This data could probably triple over the second decade of the 21th-century, with an estimated 175 million children, each year, being affected by natural disasters (46). According to the research of the Population Reference Bureau, during the 2004 Indian Ocean tsunami, women and children were more likely to suffer physical and psychological problems than men were (47). Moreover, many studies indicate that children, especially those under 8 years of age, are especially vulnerable to psychological and mental health problems following disasters (48–53). The most common symptoms and diagnoses consist of anxiety disorders, such as PTSD, panic, and phobias. Depression, acute stress reactions, adjustment disorder, and even schizophrenia, has also been reported in child survivors (54, 55). The reasons why children are especially vulnerable, mentally, to disaster may be explained through their lack of understanding about the situation, whilst they may also feel less able to control the events and be less equipped to cope with difficult situations (7).

Among adults, in direct contrast to some commonly held ideas that getting old is related to increased dependency, ailment, incapacity, loss of self-control, and social isolation that may make older people more prone to being vulnerable to disaster and life crisis, studies have discovered that older people tend to be more immune to depression (56), substance use (57), and less consistently, PTSD (13) after traumatic events. In one study conducted by Rafiey et al. (58), 324 earthquake survivors were asked to complete a Mental Health Continuum-Short Form questionnaire to investigate the long-term effect of the earthquake. A one-way multivariate analysis of covariance (MANCOVA) using SPSS was used in the data analysis (58), with the findings demonstrating a higher level of positive mental health among elderly earthquake survivors than their younger counterparts. This suggests that advancing age, *per se*, does not contribute to increased vulnerability. This result could be caused by older people's knowledge and experience in developing coping skills in post disasters (58). In addition, some studies discovered that middle-aged adults are in general, at greater risk of developing psychopathological issues after being exposed to disasters (7). Cherry et al. (59) explained this phenomenon with the burden hypothesis: people at middle age are mentally more affected by disasters than other age groups “because of their role as the economic provider with asocial and financial responsibilities for their families. For some, dual responsibilities associated with caring for dependent children and elderly parents may double the perceived burden” (59) (p. 189).

### Ethnicity

In the fourth edition of *Developmental-Behavioral Pediatrics* (60) ethnicity or ethnic group is defined as the membership of a culturally determined group or classifies by their unique cultural attributes, such as language and beliefs, usually in the context of a larger dominant society. They are frequently, but not necessarily, ranked within their societies (61). In a great many studies, ethnicity and status were proved to be one of the pre-disaster factors that have been related to mental health and resilience after disasters (7, 13, 62–64).

The study of mental health and resilience after disasters in minority groups has grown rapidly (65, 66). However, few researchers have compared African Americans, Anglos, and Latinos together, making the National Co-Morbidity Study (67) an important exception. The results of the study found that the lifetime rate of PTSD (7.8%) did not show a significant difference between whites, blacks, and Hispanics. Although black people experienced fewer traumas than whites, they were more inclined to develop PTSD once they were exposed to traumatic events.

In another study, 404 residents of southern Florida were interview six months after Hurricane Andrew (62). The participants consisted of equal numbers of Hispanics, non-Hispanic blacks, and Caucasians. Ninety-seven of the Latino interviewees decided to complete the interview in Spanish, while the rest of interviews were conducted in English. The study results indicate that people from different ethnic backgrounds differed markedly in the prevalence of PTSD (62). Specifically, with 15% prevalence, Caucasian respondents displaying the lowest rate, Spanish-preferring Latinos showed the highest rate of 38%, and African Americans showed a rate of 23%, thus between these two extremes.

Other studies suggested that a high rate of psychological disorders and mental health problems, such as PTSD, have also been found among immigrants from Central American and Mexico (68). Whereas, low rates (4%) were found among victims of a severe flood in Puerto Rico (69), higher rates (32%) have been found among Latin Americans or Hispanics following disasters in Mexico (70), Chile (19%) (71), Colombia (42%) (72), and the U.S. (12%) (73). In Miquel et al. study published in 2019, they indicated that indigenous people of Americas showed convincing and highly suggestive evidence for PTSD (28). Therefore, to summarize the findings so far, some evidence indicates that survivors of different ethnic backgrounds have no significant difference in rates of post-disaster mental health problems, and some other studies suggesting that they do.

The majority of literature regarding social class and mental health examined *differential vulnerability* as one of the potential reasons why ethnic groups respond differently after disaster events (74, 75). Differential vulnerability implies that ethnic minorities are more affected by stressors (62). This view identifies that the context of which life events are experienced is important in understanding people's reactions toward stress. The most basic reasons for this are minorities' limited access to economic and social resources that might support them during traumatic periods (76). However, research has highlighted that there is more to the concept of ethnicity than simply socioeconomic status (7). The culture's unique and specific attitudes and beliefs could also play a role in ethnic group's abilities to cope with stress and trauma (65, 77, 78). Collectivism, for example, is a sense of oneness with other people, meaning the self is defined as part of a group (79), which is in contrast to individualist cultures, where relationships between members are closer, but then more distant with members outside of their own group. An extreme form of collectivism is familism (80, 81). This interconnectedness with family and friends is a valuable resource in coping with disasters, but in some cases, it may offer disadvantages as well. Kaniasty and Norris explained that, reluctance to ask for assistance could have serious implications in disaster-stricken situations where kin's supports might be depleted and insufficient (82). Moreover, familism frequently creates an acute sense of familial obligation that may cause increased stress and distress (83).

### Social Support

Social support is a complex construct with numerous definitions (84). The term often refers to the quality and function of social relationships, specifically, it means having people like family members or close friends to turn to in times of need or crisis to provide care and help promote a positive self-image. Sippel et al. (84) indicated that social support could take many forms, such as social interactions, emotional support, instrumental/material support, information/cognitive support and so on.

Numerous literatures have shown that pre-disaster social support can decrease both exposure to natural disasters and the adverse psychological effects of natural disaster exposure (85–90). The study conducted by Sasaki et al. examined if social support before disaster mitigates post-disaster depressive symptoms of older victims of the 2011 Great East Japan earthquake and tsunami (86). The survey was conducted among 2,293 participants who are more than 65 years old living in Iwanuma city two and a half years after the disaster. The results indicated that participants who received emotional and instrumental support before the disaster were significantly less likely to develop symptoms of depression after the disaster when compared with those who lacked supports (86). A similar conclusion has been found from Chan et al. (88) research among 492 survivors of Hurricane Katrina, which showed that higher levels of pre-disaster social support was related to lower mental health and psychological problems 1 year after storms.

Insufficient social support during the post-disaster period is associated with various psychological symptoms after the disaster (6, 7, 13, 26, 91), as well as disorders like PTSD (13) major depressive disorder (MDD) and prolonged grief disorder (PGD) (56). On the contrary, stronger social support resources have been related to greater resilience (18), which may function as a buffer against negative mental health effect of stressful events through influencing how people manage the situation (92). Further, available resources indicated that good social support plays a significant role in enhancing self-confidence, decreasing the likelihood of engaging in risky behaviors, such as excessive drinking and drug-taking, whilst also promoting more healthy coping strategies, such as active problem solving (93, 94).

In one study published in 2016, to investigate the predictors of recovery from disaster, victims of the 1998 Donting Lake flood who were diagnosed with PTSD in 2000 were measured for the prevalence rate of PTSD at follow-up sessions and addressing the predictive factors of PTSD after the flood (95). The results show that among the 321 participants with prior PTSD, 51 (15.89%) of the flood survivors keeps suffering from PTSD in 2013 and 2014. Moreover, people who had lost family members and friends, who were physically injured, and had poor social support or had a negative coping style were less likely to recover from PTSD (95). The logistic regression analysis displayed that the recovery from prior mental trauma was significantly related to social support, subjective support, and support utilization (95). This is in agreement with Dr. Hall's longitudinal research among survivors living in Northern Iraq, highlighting the complicated correlation between mental health symptoms and changes in social support networks among torture survivors (96). In Hall's study, the researchers evaluated if psychological distress symptoms in the survivors was associated with social resources changes 5 months later among 96 adult males and female's treatment-seeking torture survivors residing in Kurdistan, Iraq (96). The results indicated that higher mental health symptoms have a correlation with decreased social support. Moreover, the decline of social contact was associated with increased anxiety and PTSD symptoms. On the contrary, though, gaining social contact was related to decreasing depression and PTSD symptoms (96). Similar conclusion has been found from other researches, such as an investigation in children and their experience of disasters by Green et al. (52); Dalgleish's longitudinal study about a crisis support following the *MS Herald of Free Enterprise* disaster (97); and a study by Arata's team aimed at exploring the coping strategies connected to technological disasters (25). Other studies include Johnson's research about psychiatric disorders among victims of courthouse shootings (98) and North's exploration about psychiatric disorders among survivors of the Oklahoma City Bombing (30).

Many neurocognitive systems and genetic mechanisms have been involved in linking social support and human resilience (99). One explanation for this is oxytocin. Neuropeptide oxytocin is a hormone released during social events and boosts pro-social activities by improving social recognition and the sense of trust (100). Zink and Meyer-Lindenberg discovered that the anxiolytic and pro-social effects of oxytocin appear related to improved activities of the prefrontal cortex and decreased amygdala activity (101). As a result, this decline in physiological reactivity to stress, especially chronic stress, has been related to positive psychological and physical health (84). Another possible explanation is that biochemical reactions can be triggered by social support and social environment (102, 103). One example is that some studies reported that “people who inherit a specific variation of the serotonin transporter gene, i.e., SS variation, are more likely to become depressed after stressful events compared to people who inherit other variants of the serotonin transporter gene” (104) (p. 4). Additionally, Kaufman's research team found that good quality social support offered protection against depression related to stress among children who were abused, even for individual who has the SS variation of the serotonin transporter gene. Consequently, it might be possible that gene expressions can be modified by the social environment (105–107).

### Socioeconomic Status

The American Psychological Association defined socioeconomic status as the social standing or class of an individual or group. It is often measured as a combination of education, income, and occupation (108). This status has been a reliable and consistent predictor of a vast array of outcomes across one's life span, including physical and mental health (108). Through intensive research, socioeconomic status is believed to be one of the essential factors that has been associated with greater risk of psychopathological issues after disasters (7, 13, 18, 56, 57).

In a research from the World Bank and Global Facility for Disaster Reduction and Recovery (GFDRR), the authors mentioned that people around the world might depend on non-disaster aid programs, such as Medicare and unemployment insurance, to cope with effects following disasters (109). However, they emphasize that the post-disaster support organizations or programs can offer is limited, especially in developing nations. These programs are not designed or funded to offer fast improvement, as trauma events usually require, or to transfer help to victims in poverty are typically fewer than those to the upper crust (109).

The losses produced by the disaster, such as the property loss and damage, terrible living environment, disruptions in employment, education, health care access, social supports, and daily routine, will usually lead to the elevation of stress and other mental health problems such as depression and anxiety, and the majority of these losses happen when victims of disasters are displaced (110). As noted, houses or apartments of families with low SES worldwide are more likely to be vulnerable to disasters, people of low SES are more likely to be displaced following disaster events. Fothergrill and Peek suggested that many low SES people, therefore, become homeless after a disaster (111). Previous studies indicated that displacement could affect survivors' mental health from several aspects. It usually disrupts the social support systems that mediate the mental health impacts and stress following disasters (90); It alters daily routines about home, work, school, social activities and, etc., (112); Further, it may also bring new stressors when housing conditions are unsatisfactory, or evacuees feel socially isolated, insecure, or that they are discriminated (113). Research has also noticed that families with subaverage incomes, people with unreliable employment, older adult women living in poverty, have difficulties in receiving housing loans from organizations and programs (114, 115). They believe that housing and displacement is commonly an overriding problem for low SES families at post-disaster periods, which is also one of the main stressors that result in mental health problems (116).

There are multiple studies that indicate that compared to people with high SES, low-income and low SES households lack access to resources that they need after disasters, therefore, they might experience a harder time in terms of stress s than those of higher income and SES (117–120). In a study conducted by Subaiya et al. explaining findings of an assessment conducted in the Rockaway Peninsula, part of New York City, 20 days after Superstorm Sandy, the authors reported that households from low SES notably expressed higher anxiety about food than people from high SES (121). The research team also found a trend toward psychological disturbance among low SES victims, although the trend was not statistically significant (121).

Another study on victims from Hurricane Ike, which happened in September 2008, discovered that two characteristics of low SES were significantly related to their greater likelihood of mental health problems, for example depression (122). For the 658 people who had been living in the area affected by the disaster and who was interviewed months later, survivors with a lower annual household income and fewer years of education were more likely to show mental health problems (122). A similar finding could be seen in the research on people affected by the Deepwater Horizon oil spill, which occurred between April to September 2010. Fan et al. discovered that people of low SES were much more easily to show feelings and symptoms of depression and had frequent mental distress after the disaster. Two traits related to low SES: being unemployed and low annual income, played a major role in this case (123).

Various other research also indicates that, individual with low SES face more obstacles to getting aid to assist them to reconstruct home and fulfill their other needs after disaster events. The stress associated with insufficient resources may create emotional and behavioral consequences (109), therefore people of lower SES experienced higher chances of psychological and physical problems, compared with higher SES following the experience of disasters.

### Some Other Factors

Except those factors mentioned above, various other social determinants were proved by some studies that can affect mental health and resilience in the post-trauma period. For instance, previous trauma, childhood abuse, family psychiatric history, and subsequent life stress. In the study carried out by Brewin et al. (29), the meta-analyses were conducted on 14 separated risk factors for PTSD. Factors such as previous trauma, and general childhood adversity were proved to consistently predicted PTSD but to a different extent depends on the population studies and the method used. Also, factors such as psychiatric history, childhood abused experience, and reported family psychiatric history performed stronger predictive effects. Similar results were conducted by Miquel et al. in their umbrella review of risk factors for PTSD (28). In Brewin' s review, the effect size of all the risk factors operating during or after trauma such as subsequent life stress had somewhat strongest risk of PTSD (29).

### Public Policy and Practice Implications

Efficient public policies and practices conducted during the pre, peri, and post-disaster periods can improve mental health outcome to a large extent (14). The World Bank and Global Facility for Disaster Reeducation and Recovery (GFDRR) report appeals for countries to take actions, at the government level, to reduce the vulnerability and increase the resilience of people suffering from disasters (124). Moreover, Fothergill and Peek (111) indicated that governments and other organizations worldwide should cooperatively work together to improve the security and well-being of people who experienced disaster events. Governments and those larger, multinational organizations are more likely to have the resources and authority to make policies that the general public do not (111). This section reviews several actions that could be conducted prior to, during, and after traumatic events to prevent, reduce, relieve the severity of, and treat mental disorders and promote healthy recovery.

### Before and During Disaster: Anticipating and Preparing for Disasters

One significant action in pre-disaster preparation work is to avoid or minimize exposure to potential events related to disasters which can decrease the likelihood of additional stressors and mental health problems. There are several methods that local governments, communities, and organizations can adopt to reduce the possibilities and seriousness of the traumatic events. Firstly, as Fothergill and Peek (111) suggested, governments should develop policies to improve the safety of all housing, including low-income housing, and make housing affordable for people of low SES. They believe that this could involve requiring landlords to fund improvements or providing them with subsidies or other support for doing so. Secondly, when building new real estates in vulnerable areas, governments should identify the degrees of protection to be provided against specific disasters or hazards. The development of real estates in particularly vulnerable locations should be discouraged and building regulations could be modified to prevent collapse or damage to the real estate (14). McFarlane and Williams (9) pointed out, one of the reasons behind greater devastation, injury, death, and housing loss following natural disasters in low-income countries is because these regulations are not present or enforced. Also, planning disaster-ready infrastructures such as sea defense walls is effective in reducing the impact of disasters, such as flooding caused by hurricanes (125). Local governments and communities can also invest in developing warning systems and response methods that are adaptable to various disaster situations, building on data gained from previous disasters, these systems need to operate efficiently in emergencies. Goldmann and Galea (14) suggested that government can offer incentives for power and water companies to create more robust systems to avoid extended loss of electricity, heat, and running water, which can serve as additional stressors during the post-disaster period if not provided. In the case of natural disasters, to the safe evacuation of people in the affected areas can reduce casualties. If people are evacuated, or planned to be evacuated, then well-functioning shelters, equipped with appropriate supplies and staffed to respond to victims' health needs during the disaster, are essential. Moreover, Organization (8) suggested that activities before disasters such as hazard mapping and vulnerability analysis, should integrate gender consideration, for instance considering how the vulnerability impacts differently on women and men, and how gender roles and status affect disaster-relief and mental health. Additionally, disaster workers, involved in rescue and relief measures, should be trained well in advance in the field of emotional aid and basic communication skills in dealing with traumatized victims, especially children (54).

### After Disaster: Relieving Stress and Improving Mental Health Conditions

Various interventions and practices after the disaster have been developed to assist victims in different stages of the aftermath of the disaster. The intentions of preventing the development of and treating symptoms of psychopathology and improve mental health are the key focus of such interventions (14). Although individual resilience can be enhanced through personal skill training and positive interactions with families (e.g., cognitive reframing, physical fitness, couple-based intervention for PTSD) (126), effective practices as well as specific policies by the government are now seen as critical to post-disaster resilience (127).

First, government, communities, and related institutions should provide resources to guarantee a safe neighborhood with public spaces that promote exercise, affordable housing, access to healthcare and, high quality schools. These facilities might provide a significant improvement to resilience for the large number of survivors who live in destitute and dangerous communities (128, 129). Shim's research team addressed that, with the advancement in people's awareness of how social network factors impact on each individual, it could be possible to introduce societal interventions that promote resilience in a large population of individuals physically and emotionally (130). Political practices and interventions provide solutions to societal issues, such as poverty, housing and food shortage, poor education, and addressing the wealth gap which could have substantial effects on the resilience of survivors (130). Also, as stated by Norris et al. (131) (p. 162), “when problems are shared, so must be solutions.” Traumas, such as natural disasters and terrorist attacks usually destroyed several systems concurrently. Therefore, survivors are connected and rely on each other's coping techniques, especially during the post-disaster period. Governments and communities should encourage individuals with related experiences to prepare and organize solutions to help people come out of the hard times (84).

Psychological first aid (PFA), developed by the National Child Traumatic Stress Network and the National Center for PTSD, is an evidence-informed approach that consists of actions aimed at reducing initial post-disaster distress and supporting adaptive functioning that has already been taken by many governments and organizations (132, 133). PFA has three goals: ensure victim's safety and daily necessities, such as food, water, shelter, and medical supplies; relieve severe stress through addressing stress factors after traumatic events and providing stress-reduction techniques; thirdly, assist survivors to gain additional resources to help them rebuilt confidence and the feelings of control (14, 133). Some researchers have noted that PFA offers great promise, but further empirical research is needed to test the effectiveness of the approach over the long term (132).

The introduction of innovative, cost-effective interventions such as supportive text messaging, could also be taken into consideration by policymakers (134). Mobile phone technologies, for example, have been proven to have the potential to provide personalized support for patients, and can improve outcomes for many mental health issues as well as substance use disorders (135). The recent COVID-19 pandemic has had a significant psychological effect on people worldwide. Research has already highlighted that social distance, containment, and security measures have affected the relationship among people and their perception of empathy toward others (136, 137). Children, college students, and health workers are the most exposed groups who are especially vulnerable to PTSD, anxiety, depression, and other symptoms of distress (136). In Canada, Agyapong et al. (138) launched a daily supportive SMS text messaging program “Text4Hope” to test the changes in the level of stress, anxiety, and depression of subscribers after 6 weeks of exposure to the daily supportive SMS text messages during the COVID-19 pandemic. The results indicated that at the 6-week and 3-month time points, there was a statistically significant fall in mean score on the 10-item Perceived Stress Scale (PSS-10), the 9-item Patient Health Questionnaire and the 7-item Generalized Anxiety Disorder-7 (GAD-7) (138–140). Similar results could also be found in another study carried out by Agyapong et al. (141). This study evaluated the self-report of the impact of the “Text4Mood” program which delivered daily support text messages to subscribers on their mental well-being (141). Among the 894 subscribers who answered the self-report questionnaire, 38% of whom were diagnosed with a psychiatric disorder, 81.7% respondents felt the text message brought them more confidence in managing daily issues; 76.7% reported feeling more in charge of managing depression and anxiety issues, while 75.2% felt the sense of connection through the support system. Overall, 83.1% of participants felt “Text4Mood” improved their overall mental well-being (141). Agyapong et al. suggested that the supportive text message system is therefore a convenient, cost-effective, and acceptable approach for delivering population-level psychological interventions (134, 138, 141). Moreover, the program also demonstrated a significant effect in relieving anxiety and stress levels during the COVID-19 pandemic, therefore, it could be adopt by governments as a mental health intervention to the general public during, or after, the natural disasters and other emergencies (138).

## Limitations

This literature review has some important limitations. Firstly, this is a general literature search which qualitatively summarized the evidence and provide an overview of the topic rather than a systematic literature review. Data retrieval was conducted by only one researcher in a non-systematic way which did not account for the total number of studies obtained from the search, duplicates and non-relevant studies, and therefore, a Prisma diagram has not been included. Secondly, in this article, we only searched English language databases. Academic search that relies only on certain languages or from certain countries may result in failure to find many important studies published with other languages, irrespective of the research question and the avoidance of any language restrictions (142). Finally, factors such as “social support,” “previous trauma,” “childhood adversity,” or “family psychiatric history” are indeed very general. However, as noted by Shalev et al. (143) it is probably easier to assess these “simplified” predictors in the real world, and they have been successfully applied in several fields of medicine (28). Notwithstanding the limitations of the study, this review provides insightful overview of the predictive vulnerability and protective social determinants which impact on the mental health and resiliency of survivors of disasters.

## Conclusion and Future Direction

There are numerous studies in the field of mental health and resilience after the experience of disasters. Some areas related to this field may benefit most from the existing and future research. Firstly, studies that transfer their main emphasis from the prevalence of mental health problems after the disaster, which has obviously been examined extensively, to a longitudinal assessment of disaster survivors to further elucidate disorder trajectories could offer valid contributions (6, 7, 13). Since several areas in this filed remain unclear, such as the course of mental illness such as PTSD after disaster (6), these types of studies are able to help researchers understand the possible factors associated with various reasons behind mental health problems better. Moreover, it could help clarify populations that are more at risk and identify effective intervention strategies (6, 14). Secondly, the field requires studies that evaluate a broader range of psychopathological areas than the current study. Most of the studies about post-disaster mental health problems are focusing on assessed symptoms of PTSD, whilst few studies have targeted other possible post-disasters problems, such as anxiety, depression, and panic disorder (144).

Thirdly, additional researches concentrated on interventions that aim to prevent or reduce symptoms of mental illness among survivors of disasters is required (7, 144). Even though there are some interventions and treatment strategies which were deemed to be efficacious in randomized controlled experiments, effectiveness studies are in dire need for further evaluation of the curative effect of interventions on general populations with practicing clinicians (145) and their effect on preventing or reducing comorbidity such as substance use disorders (146, 147). The existing and future research may also benefit studies that investigate a wide range of potential risk factors from multiple perspectives, such as “biological and genetic characteristics that may predispose some victims to poor mental health outcomes but not others” (14) (p. 180). Additionally, studies could examine how the interaction of genetic and environmental characteristics produce diseases, such as how the change of the environment can affect our genes behave (103, 148).

In the end, as Généreux et al. noted, good governance is perhaps the single most important factor influencing the effectiveness of emergency preparedness, response, and recovery (149). With the existing research achievements, it is necessary for government and related organizations to reform policies, innovate practices, cultivate relationships, and share responsibility for ensuring the safety, health, and well-being of affected people.

## Author Contributions

WM drafted the initial manuscript. VA conceived the study and critically reviewed the initial manuscript. All authors contributed to the literature search design and approved the final draft before submission.

## Conflict of Interest

The authors declare that the research was conducted in the absence of any commercial or financial relationships that could be construed as a potential conflict of interest.

## References

[1] Organization WH. Investing in Mental Health. (2003). Availabel onlne at: https://www.who.int/mental_health/media/investing_mnh.pdf (accessed October 15, 2020).

[2] Barry MM. Addressing the determinants of positive mental health: concepts, evidence and practice. Int J Mental Health Promot. (2009) 11:4–17. 10.1080/14623730.2009.9721788

[3] Keyes CL. Mental illness and/or mental health? Investigating axioms of the complete state model of health. J Consult Clin Psychol. (2005) 73:539. 10.1037/0022-006X.73.3.539 15982151

[4] Artiga S Hinton E. Beyond health care: the role of social determinants in promoting health and health equity. Health. (2019) 20:1–13. Availabel online at: https://files.kff.org/attachment/issue-brief-beyond-health-care

[5] Allen J Balfour R Bell R Marmot M. Social determinants of mental health. Int Rev Psychiatry. (2014) 26:392–407. 10.3109/09540261.2014.928270 25137105

[6] Neria Y Nandi A Galea S. Post-traumatic stress disorder following disasters: a systematic review. Psychol Med. (2008) 38:467. 10.1017/S0033291707001353 17803838 PMC4877688

[7] Norris FH Friedman MJ Watson PJ Byrne CM Diaz E Kaniasty K. 60,000 disaster victims speak: Part I. An empirical review of the empirical literature, 1981–2001. Psychiatry. (2002) 65:207–39. 10.1521/psyc.65.3.207.20173 12405079

[8] Organization WH. Gender and Health in Disasters. (2002). Availabel online at: https://www.who.int/gender/other_health/genderdisasters.pdf (accessed October 17, 2020).

[9] McFarlane AC Williams R. Mental health services required after disasters: learning from the lasting effects of disasters. Depress Res Treatment. (2012) 2012:970194. 10.1155/2012/970194 22811897 PMC3395273

[10] Forum WE. A Decade Left. (2019). Availabel online at: https://reports.weforum.org/global-risks-report-2020/a-decade-left/ (accessed October 17, 2020).

[11] Institute II. Facts + Statistics: Global Catastrophes. (2020). Availabel online at: https://www.iii.org/fact-statistic/facts-statistics-global-catastrophes (accessed October 17, 2020).

[12] Raphael B Maguire P. Disaster Mental Health Research: Past, Present, and Future. New York, NY: Cambridge University Press (2012).

[13] Galea S Nandi A Vlahov D. The epidemiology of post-traumatic stress disorder after disasters. Epidemiol Rev. (2005) 27:78–91. 10.1093/epirev/mxi003 15958429

[14] Goldmann E Galea S. Mental health consequences of disasters. Ann Rev Public Health. (2014) 35:169–83. 10.1146/annurev-publhealth-032013-182435 24159920

[15] Moosavi S Nwaka B Akinjise I Chue P Corbett SE Greenshaw AJ. Mental health effects in primary care patients 18-months after a major wildfire in Fort McMurray: risk increased by social-demographic issues, clinical antecedents, and degree of fire exposure. Front Psychiatry. (2019) 10:683. 10.3389/fpsyt.2019.00683 31620033 PMC6760025

[16] Breslau N Kessler RC Chilcoat HD Schultz LR Davis GC Andreski P. Trauma and posttraumatic stress disorder in the community: the 1996 detroit area survey of trauma. Arch Gene Psychiatry. (1998) 55:626–32. 10.1001/archpsyc.55.7.626 9672053

[17] Norris FH Tracy M Galea S. Looking for resilience: understanding the longitudinal trajectories of responses to stress. Soc Sci Med. (2009) 68:2190–8. 10.1016/j.socscimed.2009.03.043 19403217

[18] Bonanno GA Gupta S. Resilience after disaster. Mental Health Disasters. (2009) 2009:145–60. 10.1017/CBO9780511730030.009

[19] Association AP. The Road to Resilience: What Is Resilience? (2013). Availabel online at: http://www.apa.org/helpcenter/road-resilience.aspx (accessed October 18, 2020).

[20] Momsen J. Gender and Development. London, UK: Routledge (2019). 10.4324/9781315674186

[21] Karsten J Hartman CA Smit JH Zitman FG Beekman AT Cuijpers P. Psychiatric history and subthreshold symptoms as predictors of the occurrence of depressive or anxiety disorder within 2 years. Br J Psychiatry. (2011) 198:206–12. 10.1192/bjp.bp.110.080572 21357879

[22] Grieger TA Fullerton CS Ursano RJ. Posttraumatic stress disorder, alcohol use, and perceived safety after the terrorist attack on the Pentagon. Psychiatr Serv. (2003) 54:1380–2. 10.1176/appi.ps.54.10.1380 14557524

[23] Pulcino T Galea S Ahern J Resnick H Foley M Vlahov D. Posttraumatic stress in women after the september 11 terrorist attacks in New York City. J Women Health. (2003) 12:809–20. 10.1089/154099903322447774 14588131

[24] Schuster MA Stein BD Jaycox LH Collins RL Marshall GN Elliott MN. A national survey of stress reactions after the September 11, 2001 terrorist attacks. N Engl J Med. (2001) 345:1507–12. 10.1056/NEJM200111153452024 11794216

[25] Arata CM Picou JS Johnson GD McNally TS. Coping with technological disaster: an application of the conservation of resources model to the Exxon Valdez oil spill. J Traumatic Stress. (2000) 13:23–39. 10.1023/A:1007764729337 10761172

[26] Agyapong VI Ritchie A Brown MR Noble S Mankowsi M Denga E. Long-term mental health effects of a devastating wildfire are amplified by socio-demographic and clinical antecedents in elementary and high school staff. Front Psychiatry. (2020) 11:448. 10.3389/fpsyt.2020.00448 32528323 PMC7265240

[27] Agyapong VI Juhás M Omege J Denga E Nwaka B Akinjise I. Prevalence rates and correlates of likely post-traumatic stress disorder in residents of Fort McMurray 6 months after a wildfire. Int J Mental Health Addict. (2019) 2019:1–19. 10.1007/s11469-019-00096-z

[28] Tortella-Feliu M Fullana MA Pérez-Vigil A Torres X Chamorro J Littarelli SA. Risk factors for posttraumatic stress disorder: an umbrella review of systematic reviews and meta-analyses. Neurosci Biobehav Rev. (2019) 107:154–65. 10.1016/j.neubiorev.2019.09.013 31520677

[29] Brewin CR Andrews B Valentine JD. Meta-analysis of risk factors for posttraumatic stress disorder in trauma-exposed adults. J Consul Clin Psychol. (2000) 68:748. 10.1037/0022-006X.68.5.748 11068961

[30] North CS Tivis L McMillen JC Pfefferbaum B Cox J Spitznagel EL. Coping, functioning, and adjustment of rescue workers after the Oklahoma City bombing. J Traumatic Stress. (2002) 15:171–5. 10.1023/A:1015286909111 12092908

[31] Irmansyah I Dharmono S Maramis A Minas H. Determinants of psychological morbidity in survivors of the earthquake and tsunami in Aceh and Nias. Int J Mental Health Syst. (2010) 4:8. 10.1186/1752-4458-4-8 20423505 PMC2873571

[32] Agyapong VI Hrabok M Juhas M Omeje J Denga E Nwaka B. Prevalence rates and predictors of generalized anxiety disorder symptoms in residents of Fort McMurray six months after a wildfire. Front Psychiatry. (2018) 9:345. 10.3389/fpsyt.2018.00345 30108527 PMC6079280

[33] Kessler RC McGonagle KA Zhao S Nelson CB Hughes M Eshleman S. Lifetime and 12-month prevalence of DSM-III-R psychiatric disorders in the United States: results from the national comorbidity survey. Arch Gene Psychiatry. (1994) 51:8–19. 10.1001/archpsyc.1994.03950010008002 8279933

[34] Neumayer E Plümper T. The gendered nature of natural disasters: the impact of catastrophic events on the gender gap in life expectancy, 1981–2002. Ann Assoc Am Geograph. (2007) 97:551–66. 10.1111/j.1467-8306.2007.00563.x

[35] Moreno J Shaw D. Women's empowerment following disaster: a longitudinal study of social change. Nat Hazards. (2018) 92:205–24. 10.1007/s11069-018-3204-4

[36] Nature IUFCO. How Natural Disasters Affect Women. (2009). Availabel online at: https://www.iucn.org/content/how-natural-disasters-affect-women (accessed October 19, 2020).

[37] Cross IFOR Societies RC. A Practical Guide to Gender-Sensitive Approaches for Disaster Management. Geneva: Federation of Red Cross and Red Crescent Societies (2010).

[38] Shah SA. Gender and building homes in disaster in Sindh, Pakistan. Gender Dev. (2012) 20:249–64. 10.1080/13552074.2012.687222

[39] Bradshaw S Fordham M. Double Disaster: Disaster Through a Gender Lens, in Hazards, Risks and Disasters in Society Milton, MA: Elsevier (2015). 10.1016/B978-0-12-396451-9.00014-7

[40] Enarson E Fothergill A Peek L. Gender and Disaster: Foundations and Directions, in Handbook of Disaster Research. New York, NY: Springer (2007). 10.1007/978-0-387-32353-4_8

[41] Pittaway E Bartolomei L Rees S. Gendered dimensions of the 2004 tsunami and a potential social work response in post-disaster situations. Int Soc Work. (2007) 50:307–19. 10.1177/0020872807076042

[42] Women U. Time to Act on Gender, Climate Change and Disaster Risk Reduction. (2016). Availabel online at: https://asiapacific.unwomen.org/en/digital-library/publications/2016/11/time-to-act (accessed October 20, 2020).

[43] Orui M Sato Y Tazaki K Kawamura I Harada S Hayashi M. Delayed increase in male suicide rates in tsunami disaster-stricken areas following the great east japan earthquake: a three-year follow-up study in Miyagi Prefecture. Tohoku J Experi Med. (2015) 235:215–22. 10.1620/tjem.235.215 25765170

[44] Guiney R. Farming suicides during the victorian drought: 2001–2007. Austr J Rural Health. (2012) 20:11–5. 10.1111/j.1440-1584.2011.01244.x 22250871

[45] Penrose A Takaki M. Children's rights in emergencies and disasters. Lancet. (2006) 367:698–9. 10.1016/S0140-6736(06)68272-X 16503472

[46] Codreanu TA Celenza A Jacobs I. Does disaster education of teenagers translate into better survival knowledge, knowledge of skills, and adaptive behavioral change? A systematic literature review. Prehospital Disaster Med. (2014) 29:629. 10.1017/S1049023X14001083 25327571

[47] Bureau PR. Disaster Risk and Vulnerability: The Role and Impact of Population and Society. (2011). Availabel online at: https://www.prb.org/disaster-risk/ (accessed October 30, 2020).

[48] Brown MR Agyapong V Greenshaw AJ Cribben I Brett-MacLean P Drolet J. After the Fort McMurray wildfire there are significant increases in mental health symptoms in grade 7–12 students compared to controls. BMC Psychiatry. (2019) 19:18. 10.1186/s12888-019-2074-y 30630501 PMC6329184

[49] Brown MR Agyapong V Greenshaw AJ Cribben I Brett-MacLean P Drolet J. Significant PTSD and other mental health effects present 18 months after the Fort Mcmurray wildfire: findings from 3,070 grades 7–12 students. Front Psychiatry. (2019) 10:623. 10.3389/fpsyt.2019.00623 31543839 PMC6728415

[50] Fairbrother K Warn J. Workplace dimensions, stress and job satisfaction. J Manage Psychol. (2003) 18:1. 10.1108/02683940310459565

[51] Pfefferbaum A Sullivan EV Rosenbloom MJ Mathalon DH Lim KO. A controlled study of cortical gray matter and ventricular changes in alcoholic men over a 5-year interval. Arch Gene Psychiatry. (1998) 55:905–12. 10.1001/archpsyc.55.10.905 9783561

[52] Green BL Korol M Grace MC Vary MG Leonard AC Gleser GC. Children and disaster: age, gender, and parental effects on PTSD symptoms. J Am Acad Child Adolesc Psychiatry. (1991) 30:945–51. 10.1097/00004583-199111000-00012 1757444

[53] Goenjian AK Pynoos RS Steinberg AM Najarian LM Asarnow JR Karayan I. Psychiatric comorbidity in children after the 1988: earthquake in Armenia. J Am Acad Child Adolesc Psychiatry. (1995) 34:1174–84. 10.1097/00004583-199509000-00015 7559312

[54] Kar N. Psychological impact of disasters on children: review of assessment and interventions. World J Pediatr. (2009) 5:5–11. 10.1007/s12519-009-0001-x 19172325

[55] Math SB Tandon S Girimaji SC Benegal V Kumar U Hamza A. Psychological impact of the tsunami on children and adolescents from the andaman and nicobar islands. Primary Care Compan J Clin Psychiatry. (2008) 10:31. 10.4088/PCC.v10n0106 18311419 PMC2249818

[56] Maguen S Neria Y Conoscenti LM Litz BT. Depression and prolonged grief in the wake of disasters. In: Neria Y Galea S Norris FH editors. Mental Health and Disasters. Cambridge: Cambridge University Press (2012). p. 116–30.

[57] van der Velden P Kleber R. Substance Use and Misuse After Disasters, in Mental Health and Disasters. Cambridge: Cambrindge University Press. (2009). 10.1017/CBO9780511730030.006

[58] Rafiey H Momtaz YA Alipour F Khankeh H Ahmadi S Khoshnami MS. Are older people more vulnerable to long-term impacts of disasters? Clin Intervent Aging. (2016) 11:1791. 10.2147/CIA.S122122 27994445 PMC5153288

[59] Cherry KE Brown JS Marks LD Galea S Volaufova J Lefante C. Longitudinal assessment of cognitive and psychosocial functioning after Hurricanes Katrina and Rita: exploring disaster impact on middle-aged, older, and oldest-old adults. J Appl Biobehav Res. (2011) 16:187–211. 10.1111/j.1751-9861.2011.00073.x 23526570 PMC3604896

[60] Harkness S Keefer CH Super CM. Culture and Ethnicity, in Developmental-Behavioral Pediatrics.Milton, MA: Elsevier (2009).

[61] Berreman GD. Inequality: comparative aspects. Int Encyclopedia Soc Behav Sci. (2015) 11:894–8. 10.1016/B978-0-08-097086-8.12093-8

[62] Perilla JL Norris FH Lavizzo EA. Ethnicity, culture, and disaster response: identifying and explaining ethnic differences in PTSD six months after Hurricane Andrew. J Soc Clin Psychol. (2002) 21:20–45. 10.1521/jscp.21.1.20.22404

[63] March JS Amaya-Jackson L Terry R Costanzo P. Posttraumatic symptomatology in children and adolescents after an industrial fire. J Am Acad Child Adolesc Psychiatry. (1997) 36:1080–8. 10.1097/00004583-199708000-00015 9256587

[64] La Greca AM Silverman WK Vernberg EM Prinstein MJ. Symptoms of posttraumatic stress in children after Hurricane Andrew: a prospective study. J Consult Clin Psychol. (1996) 64:712. 10.1037/0022-006X.64.4.712 8803361

[65] Allen IM. PTSD among African Americans. In: Marsella AJ Friedman MJ Gerrity ET Scurfield RM editors. Ethnocultural Aspects of Posttraumatic Stress Disorder: Issues, Research, and Clinical Applications. American Psychological Association (1996). p. 209–38. 10.1037/10555-008

[66] Hough RL Canino GJ Abueg FR Gusman FD. PTSD and related stress disorders among Hispanics. In: Marsella AJ Friedman MJ Gerrity ET Scurfield RM editors. Ethnocultural Aspects of Posttraumatic Stress Disorder: Issues, Research, and Clinical Applications. Washington, DC: American Psychological Association. (1996). p. 301–38.

[67] Kessler RC Sonnega A Bromet E Hughes M Nelson CB. Posttraumatic stress disorder in the national comorbidity survey. Arch Gene Psychiatry. (1995) 52:1048–60. 10.1001/archpsyc.1995.03950240066012 7492257

[68] Cervantes RC de Snyder VNS Padilla AM. Posttraumatic stress in immigrants from Central America and Mexico. Psychiatr Serv. (1989) 40:615–9. 10.1176/ps.40.6.615 2737629

[69] Canino G Bravo M Rubio-Stipec M Woodbury M. The impact of disaster on mental health: prospective and retrospective analyses. Int J Mental Health. (1990) 19:51–69. 10.1080/00207411.1990.11449153

[70] de la Fuente R. The mental health consequences of the 1985 earthquakes in Mexico. Int J Mental Health. (1990) 19:21–9. 10.1080/00207411.1990.11449159

[71] Durkin ME. Major depression and post-traumatic stress disorder following the Coalinga and Chile earthquakes: a cross-cultural comparison. J Soc Behav Personal. (1993) 8:405.

[72] Lima BR Pai S Santacruz H Lozano J. Psychiatric disorders among poor victims following a major disaster: Armero, Columbia. J Nervous Mental Dis. (1991) 179:420–7. 10.1097/00005053-199107000-00006 1869871

[73] Hough R Vega W Valle R Kolody B Griswold del Castillo R Tarke H. The prevalence of symptoms of posttraumatic stress disorder in the aftermath of the San Ysidro massacre. J Traumatic Stress. (1990) 3:71–92. 10.1002/jts.2490030106

[74] Dohrenwend BP Dohrenwend BS. Social Status and Psychological Disorder: A Causal Inquiry. New York, NY: John Wiley & Sons (1969). 10.2307/3349184

[75] Kessler RC. A disaggregation of the relationship between socioeconomic status and psychological distress. Am Sociol Rev. (1982) 47:752–64. 10.2307/2095211 7171158

[76] Kaniasty K Norris FH. In search of altruistic community: patterns of social support mobilization following Hurricane Hugo. Am J Commun Psychol. (1995) 23:447–77. 10.1007/BF02506964 8546107

[77] Anderson LP. Acculturative stress: a theory of relevance to Black Americans. Clin Psychol Rev. (1991) 11:685–702. 10.1016/0272-7358(91)90126-F

[78] Cervantes RC Castro FG. Stress, coping, and Mexican American mental health: a systematic review. Hispanic J Behav Sci. (1985) 7:1–73. 10.1177/07399863850071001

[79] Hui CH Triandis HC. Individualism-collectivism: a study of cross-cultural researchers. J Cross-Cult Psychol. (1986) 17:225–48. 10.1177/0022002186017002006

[80] Sabogal F Marín G Otero-Sabogal R Marín BV Perez-Stable EJ. Hispanic familism and acculturation: what changes and what doesn't? Hispanic J Behav Sci. (1987) 9:397–412. 10.1177/0739986387009400318791899

[81] Triandis HC. Some dimensions of intercultural variation and their implications for community psychology. J Commun Psychol. (1983) 11:285–302. 10.1002/1520-6629(198310)11:4&lt;285::AID-JCOP2290110403&gt;3.0.CO;2-8

[82] Kaniasty K Norris FH. A test of the social support deterioration model in the context of natural disaster. J Personal Soc Psychol. (1993) 64:395. 10.1037/0022-3514.64.3.395 8468668

[83] Mirowsky J., Ross CE. Mexican culture and its emotional contradictions. J Health Soc Behav. (1984) 1984:2–13. 10.2307/2136700 6725922

[84] Sippel LM Pietrzak RH Charney DS Mayes LC Southwick SM. How does social support enhance resilience in the trauma-exposed individual? Ecol Soc. (2015) 20:10. 10.5751/ES-07832-200410

[85] Sasaki Y Tsuji T Koyama S Tani Y Saito T Kondo K. Neighborhood ties reduced depressive symptoms in older disaster survivors: Iwanuma study, a natural experiment. Int J Environ Res Publ Health. (2020) 17:337. 10.3390/ijerph17010337 31947798 PMC6981381

[86] Sasaki Y Aida J Tsuji T Koyama S Tsuboya T Saito T. Pre-disaster social support is protective for onset of post-disaster depression: prospective study from the Great East Japan Earthquake & Tsunami. Sci Rep. (2019) 9:1–10. 10.1038/s41598-019-55953-7 31857658 PMC6923367

[87] Hikichi H Aida J Tsuboya T Kondo K Kawachi I. Can community social cohesion prevent posttraumatic stress disorder in the aftermath of a disaster? A natural experiment from the 2011 Tohoku earthquake and tsunami. Am J Epidemiol. (2016) 183:902–10. 10.1093/aje/kwv335 27026337 PMC4867157

[88] Chan CS Lowe SR Weber E Rhodes JE. The contribution of pre-and postdisaster social support to short-and long-term mental health after Hurricanes Katrina: a longitudinal study of low-income survivors. Soc Sci Med. (2015) 138:38–43. 10.1016/j.socscimed.2015.05.037 26046725

[89] Matsumoto S Yamaoka K Inoue M Muto S. Social ties may play a critical role in mitigating sleep difficulties in disaster-affected communities: a cross-sectional study in the Ishinomaki area, Japan. Sleep. (2014) 37:137–45. 10.5665/sleep.3324 24470703 PMC3902873

[90] Lowe SR Chan CS Rhodes JE. Pre-hurricane perceived social support protects against psychological distress: a longitudinal analysis of low-income mothers. J Consult Clin Psychol. (2010) 78:551. 10.1037/a0018317 20658811 PMC3618961

[91] Agyapong VI Juhás M Brown MR Omege J Denga E Nwaka B. Prevalence rates and correlates of probable major depressive disorder in residents of Fort McMurray 6 months after a wildfire. Int J Mental Health Addict. (2019) 17:120–36. 10.1007/s11469-018-0004-8

[92] Guay S Billette V Marchand A. Exploring the links between posttraumatic stress disorder and social support: processes and potential research avenues. J Traumatic Stress. (2006) 19:327–38. 10.1002/jts.20124 16788995

[93] Holahan CJ Moos RH Holahan CK Brennan PL. Social support, coping, and depressive symptoms in a late-middle-aged sample of patients reporting cardiac illness. Health Psychol. (1995) 14:152. 10.1037/0278-6133.14.2.152 7789351

[94] Rozanski A Blumenthal JA Kaplan J. Impact of psychological factors on the pathogenesis of cardiovascular disease and implications for therapy. Circulation. (1999) 99:2192–217. 10.1161/01.CIR.99.16.2192 10217662

[95] Dai W Wang J Kaminga AC Chen L Tan H Lai Z. Predictors of recovery from post-traumatic stress disorder after the dongting lake flood in China: a 13–14 year follow-up study. BMC Psychiatry. (2016) 16:1–9. 10.1186/s12888-016-1097-x 27825328 PMC5101704

[96] Hall BJ Bonanno GA Bolton PA Bass JK. A longitudinal investigation of changes to social resources associated with psychological distress among Kurdish torture survivors living in Northern Iraq. J Traumatic Stress. (2014) 27:446–53. 10.1002/jts.21930 25079708

[97] Dalgleish T Joseph S Thrasher S Tranah T Yule W. Crisis support following the Herald of free-enterprise disaster: a longitudinal perspective. J Traumatic Stress. (1996) 9:833–45. 10.1002/jts.2490090411 8902749

[98] Johnson SD North CS Smith EM. Psychiatric disorders among victims of a courthouse shooting spree: a three-year follow-up study. Commun Mental Health J. (2002) 38:181–94. 10.1023/A:1015269521969 12046673

[99] Ozbay F Fitterling H Charney D Southwick S. Social support and resilience to stress across the life span: a neurobiologic framework. Curr Psychiatry Rep. (2008) 10:304. 10.1007/s11920-008-0049-7 18627668

[100] Ross HE Young LJ. Oxytocin and the neural mechanisms regulating social cognition and affiliative behavior. Front Neuroendocrinol. (2009) 30:534–47. 10.1016/j.yfrne.2009.05.004 19481567 PMC2748133

[101] Zink CF Meyer-Lindenberg A. Human neuroimaging of oxytocin and vasopressin in social cognition. Hormones Behav. (2012) 61:400–9. 10.1016/j.yhbeh.2012.01.016 22326707 PMC3312952

[102] Nestler EJ. Stress makes its molecular mark. Nature. (2012) 490:171–2. 10.1038/490171a 23060173 PMC4858713

[103] Toyokawa S Uddin M Koenen KC Galea S. How does the social environment ‘get into the mind'? Epigenetics at the intersection of social and psychiatric epidemiology. Soc Sci Med. (2012) 74:67–74. 10.1016/j.socscimed.2011.09.036 22119520 PMC3246041

[104] Karg K Burmeister M Shedden K Sen S. The serotonin transporter promoter variant (5-HTTLPR), stress, and depression meta-analysis revisited: evidence of genetic moderation. Arch Gene Psychiatry. (2011) 68:444–54. 10.1001/archgenpsychiatry.2010.189 21199959 PMC3740203

[105] Davidson RJ McEwen BS. Social influences on neuroplasticity: stress and interventions to promote well-being. Nat Neurosci. (2012) 15:689–95. 10.1038/nn.3093 22534579 PMC3491815

[106] McEwen BS Getz L. Lifetime experiences, the brain and personalized medicine: an integrative perspective. Metabolism. (2013) 62:S20–6. 10.1016/j.metabol.2012.08.020 23009787

[107] Yang BZ Zhang H Ge W Weder N Douglas-Palumberi H Perepletchikova F. Child abuse and epigenetic mechanisms of disease risk. Am J Prevent Med. (2013) 44:101–7. 10.1016/j.amepre.2012.10.012 23332324 PMC3758252

[108] Association AP. Socioeconomic Status. (2020). Availabel online at: https://www.apa.org/topics/socioeconomic-status (accessed October 25, 2020).

[109] Administration SAAMHS. Disaster Technical Assistance Center Supplemental Research Bulletin. Greater Impact: How Disasters Affect People of Low Socioeconomic Status. (2017). Availabel online at: https://www.samhsa.gov/sites/default/files/dtac/srb-low-ses_2.pdf (accessed October 27, 2020).

[110] Fussell E Lowe SR. The impact of housing displacement on the mental health of low-income parents after Hurricane Katrina. Soc Sci Med. (2014) 113:137–44. 10.1016/j.socscimed.2014.05.025 24866205 PMC4096953

[111] Fothergill A Peek LA. Poverty and disasters in the United States: a review of recent sociological findings. Nat Hazards. (2004) 32:89–110. 10.1023/B:NHAZ.0000026792.76181.d9

[112] Abramson DM Stehling-Ariza T Park YS Walsh L Culp D. Measuring individual disaster recovery: A socioecological framework. Disaster Med Public Health Prep. (2010) 4(Suppl. 1):S46–54. 10.1001/dmp.2010.14 23105035

[113] Weems CF Watts SE Marsee MA Taylor LK Costa NM Cannon MF. The psychosocial impact of Hurricane Katrina: contextual differences in psychological symptoms, social support, and discrimination. Behav Res Therapy. (2007) 45:2295–306. 10.1016/j.brat.2007.04.013 17568560

[114] Bolin R. Household and Community Recovery after Earthquakes Boulder, NV: University of Colorado: Institute of Behavioral Science (1993).

[115] Childers CD. Elderly female-headed households in the disaster loan process. Int J Mass Emerg Disasters. (1999) 17:99–110.

[116] Quarantelli EL. Technological and Natural Disasters and Ecological Problems: Similarities and Differences in Planning for and Managing Them Newark, NJ: Disaster Research Center (1993).

[117] Stanford L. The Northridge Earthquake: Vulnerability and Disaster. London: Taylor & Francis (1998).

[118] Bolin RC Bolton PA. Race, Religion, and Ethnicity in Disaster Recovery. Paper 88. FMHI Publications (1986). Availabel online at: http://scholarcommons.usf.edu/fmhi_pub/88

[119] Hewitt K. Regions of Risk: A Geographical Introduction to Disasters. London: Routledge (2014).

[120] Tierney K. The whittier narrows, California earthquake of october 1, 1987—social aspects. Earthq Spectra. (1988) 4:11–23. 10.1193/1.1585460

[121] Subaiya S Moussavi C Velasquez A Stillman J. A rapid needs assessment of the rockaway Peninsula in New York City after Hurricane Sandy and the relationship of socioeconomic status to recovery. Am J Publ Health. (2014) 104:632–8. 10.2105/AJPH.2013.301668 PMC4025720 24524494

[122] Tracy M Norris FH Galea S. Differences in the determinants of posttraumatic stress disorder and depression after a mass traumatic event. Depress Anxiety. (2011) 28:666–75. 10.1002/da.20838 21618672 PMC3145817

[123] Fan AZ Prescott MR Zhao G Gotway CA Galea S. Individual and community-level determinants of mental and physical health after the deepwater Horizon oil spill: findings from the Gulf States population survey. J Behav Health Serv Res. (2015) 42:23–41. 10.1007/s11414-014-9418-7 25124651

[124] Hallegatte S Rozenberg J. Climate change through a poverty lens. Nat Clim Change. (2017) 7:250–6. 10.1038/nclimate3253

[125] Klinenberg EJJ. Adaptation. New Yorker. (2013) 7:32–7. Availabel online at: https://www.newyorker.com/magazine/2013/01/07/adaptation-eric-klinenberg

[126] Monson CM Fredman SJ Macdonald A Pukay-Martin ND Resick PA Schnurr PPJJ. Effect of cognitive-behavioral couple therapy for PTSD: a randomized controlled trial. JAMA. (2012) 308:700–9. 10.1001/jama.2012.9307 22893167 PMC4404628

[127] Southwick SM Charney DSJS. The science of resilience: implications for the prevention and treatment of depression. Science. (2012) 338:79–82. 10.1126/science.1222942 23042887

[128] Hobfoll SEJAP. The influence of culture, community, and the nested-self in the stress process: advancing conservation of resources theory. Appl Psychol. (2001) 50:337–421. 10.1111/1464-0597.00062

[129] Hall JS Zautra AJ. Indicators of community resilience: what are they, why bother? In: Reich JW Zautra AJ Hall JS editors. Handbook of Adult Resilience. New York, NY: The Guilford Press (2010). p. 350–71.

[130] Shim R Koplan C Langheim FJ Manseau MW Powers RA Compton MTJPA. The social determinants of mental health: an overview and call to action. Psychiatr Ann. (2014) 44:22–6. 10.3928/00485713-20140108-04

[131] Norris FH Sherrieb K Pfefferbaum B. Community resilience: concepts, assessment, and implications for intervention. In: Southwick SM Litz BT Charney D Friedman MJeditors. Resilience and Mental Health: Challenges Across the Lifespan. New York, NY: Cambridge University Press (2011). p. 162–75. 10.1017/CBO9780511994791.013

[132] Bryant R Litz BJM. Mental Health Treatments in the Wake of Disaster. Cambridge: Cambridge University Press (2009). 10.1017/CBO9780511730030.019

[133] Ruzek JI Brymer MJ Jacobs AK Layne CM Vernberg EM Watson P. Psychological first aid. J Mental Health Counsel. (2007) 29:17–49. 10.17744/mehc.29.1.5racqxjueafabgwp

[134] Agyapong VI Ahern S McLoughlin DM Farren CKJJ. Supportive text messaging for depression and comorbid alcohol use disorder: single-blind randomised trial. J Affect Disord. (2012) 141:168–76. 10.1016/j.jad.2012.02.040 22464008

[135] Agyapong VIO Farren CK McLoughlin DM. Mobile phone text message interventions in psychiatry-what are the possibilities? Curr Psychiatry Rev. (2011) 7:50–6. 10.2174/157340011795945847

[136] Saladino V Algeri D Auriemma V. The psychological and social impact of Covid-19: new perspectives of well-being. Front Psychol. (2020) 11:2550. 10.3389/fpsyg.2020.577684 33132986 PMC7561673

[137] Mrklas K Shalaby R Hrabok M Gusnowski A Vuong W Surood S. Prevalence of perceived stress, anxiety, depression, and obsessive-compulsive symptoms in health care workers and other workers in alberta during the COVID-19 pandemic: cross-sectional survey. JMIR Ment Health. (2020) 7:e22408. 10.2196/22408 32915764 PMC7527165

[138] Agyapong VIO Hrabok M Vuong W Shalaby R Noble JM Gusnowski A. Changes in stress, anxiety, and depression levels of subscribers to a daily supportive text message program (Text4Hope) During the COVID-19 pandemic: cross-sectional survey study. JMIR Ment Health. (2020) 7:e22423. 10.2196/22423 33296330 PMC7752184

[139] Agyapong VI Hrabok M Shalaby R Vuong W Noble JM Gusnowski A. Text4Hope: receiving daily supportive text messages for 3 months during the COVID-19 pandemic reduces stress, anxiety, and depression. Disaster Med Public Health Prep. (2021) 8:1–5. 10.1017/dmp.2021.27 33551009 PMC7985636

[140] Agyapong VI Shalaby R Hrabok M Vuong W Noble JM Gusnowski A. Mental health outreach via supportive text messages during the CoViD-19 pandemic: improved mental health and reduced suicidal ideation after six weeks in subscribers of Text4Hope compared to a control population. Int J Environ Res Public Health. (2021) 18:2157. 10.3390/ijerph18042157 33672120 PMC7927101

[141] Agyapong VI Mrklas K Juhás M Omeje J Ohinmaa A Dursun SM. Cross-sectional survey evaluating Text4Mood: mobile health program to reduce psychological treatment gap in mental healthcare in Alberta through daily supportive text messages. BMC Psychiatry. (2016) 16:378. 10.1186/s12888-016-1104-2 27821096 PMC5100254

[142] Pilkington K Boshnakova A Clarke M Richardson J Medicine C. No language restrictions in database searches: what does this really mean? J Altern Complement Med. (2005) 11:205–7. 10.1089/acm.2005.11.205 15750383

[143] Shalev AY Gevonden M Ratanatharathorn A Laska E Van Der Mei WF Qi W. Estimating the risk of PTSD in recent trauma survivors: results of the International Consortium to Predict PTSD (ICPP). World Psychiatry. (2019) 18:77–87. 10.1002/wps.20608 30600620 PMC6313248

[144] McFarlane A Van Hooff M Goodhew F. Anxiety Disorders and PTSD. Cambridge: Cambridge University Press (2009).

[145] Levitt J Hoagwood K Greene L Rodriguez J Radigan M. Mental Health Care for Children in the Wake of Disasters. Mental Health and Disasters. New York, NY: Cambridge University Press (2009).

[146] Brady KT Killeen TK Brewerton T Lucerini S. Comorbidity of psychiatric disorders and posttraumatic stress disorder. J Clin Psychiatry. (2000) 61(Suppl. 7):22–32. 10795606

[147] Difede J Cukor J. Evidence-based long-term treatment of mental health consequences of disasters among adults. In: Neria Y Galea S Norris FH editors. Mental Health and Disasters. Cambridge: Cambridge University Press (2012). p. 336–49.

[148] Dick DM. Gene-environment interaction in psychological traits and disorders. Ann Rev Clin Psychol. (2011) 7:383–409. 10.1146/annurev-clinpsy-032210-104518 21219196 PMC3647367

[149] Généreux M Lafontaine M Eykelbosh A Health P. From science to policy and practice: a critical assessment of knowledge management before, during, and after environmental public health disasters. Int J Environ Res Public Health. (2019) 16:587. 10.3390/ijerph16040587 30781625 PMC6407109

